# The Impact of Corticosteroids on Human Airway Smooth Muscle Contractility and Airway Hyperresponsiveness: A Systematic Review

**DOI:** 10.3390/ijms232315285

**Published:** 2022-12-04

**Authors:** Luigino Calzetta, Alfredo Chetta, Marina Aiello, Elena Pistocchini, Paola Rogliani

**Affiliations:** 1 Respiratory Disease and Lung Function Unit, Department of Medicine and Surgery, University of Parma, 43126 Parma, Italy; 2 Unit of Respiratory Medicine, Department of Experimental Medicine, University of Rome “Tor Vergata”, 00133 Rome, Italy

**Keywords:** AHR, airway smooth muscle, asthma, corticosteroid, genomic, non-genomic

## Abstract

Classically, the effects elicited by corticosteroids (CS) are mediated by the binding and activation of cytosolic glucocorticoid receptors (GR). However, several of the non-genomic effects of CS seem to be mediated by putative non-classic membrane receptors characterized by pharmacological properties that are different from those of classic cytosolic GR. Since pre-clinical findings suggest that inhaled CS (ICS) may also regulate the bronchial contractile tone via putative CS membrane-associate receptors, the aim of this review was to systematically report and discuss the impact of CS on human airway smooth muscle (ASM) contractility and airway hyperresponsiveness (AHR). Current evidence indicates that CS have significant genomic/non-genomic beneficial effects on human ASM contractility and AHR, regardless of their anti-inflammatory effects. CS are effective in reducing either the expression, synthesis or activity of α-actin, CD38, inositol phosphate, myosin light chain kinase, and ras homolog family member A in response to several pro-contractile stimuli; overall these effects are mediated by the genomic action of CS. Moreover, CS elicited a strong bronchorelaxant effect via the rapid activation of the Gsα–cyclic-adenosine-monophosphate–protein-kinase-A pathway in hyperresponsive airways. The possibility of modulating the dose of the ICS in a triple ICS/long-acting β_2_-adrenoceptor agonist/long-acting muscarinic antagonist fixed-dose combination supports the use of a Triple MAintenance and Reliever Therapy (TriMART) in those asthmatic patients at Step 3–5 who may benefit from a sustained bronchodilation and have been suffering from an increased parasympathetic tone.

## 1. Introduction

Generally, the effects elicited by corticosteroids (CS) are generally mediated by the binding and activation of cytosolic glucocorticoid receptors (GR) that, in turn, translocate towards the nucleus, interact with glucocorticoid response elements (GRE), and ultimately elicit genomic effects that modulate protein expression [1,2]. Indeed, such a complex cascade requires a prolonged onset of action to activate/inhibit genomic processes for CS and also other steroid hormones [1,3].

However, a wide range of non-genomic effects of CS seems to be mediated by putative non-classic membrane receptors characterized by pharmacological properties that are different from those of classic cytosolic GR [3]. Interestingly, the rapid, almost-immediate, non-genomic effects of CS regulate several signaling processes leading to effects on intracellular calcium mobilization and homeostasis, reactive oxygen and nitrogen species, inflammatory and apoptotic pathways, and skeletal and smooth muscle function [1]. In this regard, current pre-clinical evidence suggests that in human isolated airways inhaled CS (ICS) may also regulate the bronchial contractile tone via putative CS membrane-associate receptors [4].

Therefore, the aim of this review was to systematically report and discuss the impact of CS on human airway smooth muscle (ASM) contractility and airway hyperresponsiveness (AHR).

## 2. Methods

### 2.1. Review Question

The question of this systematic review was to assess the impact of CS on human ASM contractility and AHR that was not related to the anti-inflammatory effects of CS.

### 2.2. Search Strategy and Study Eligibility

**Figure 1:**
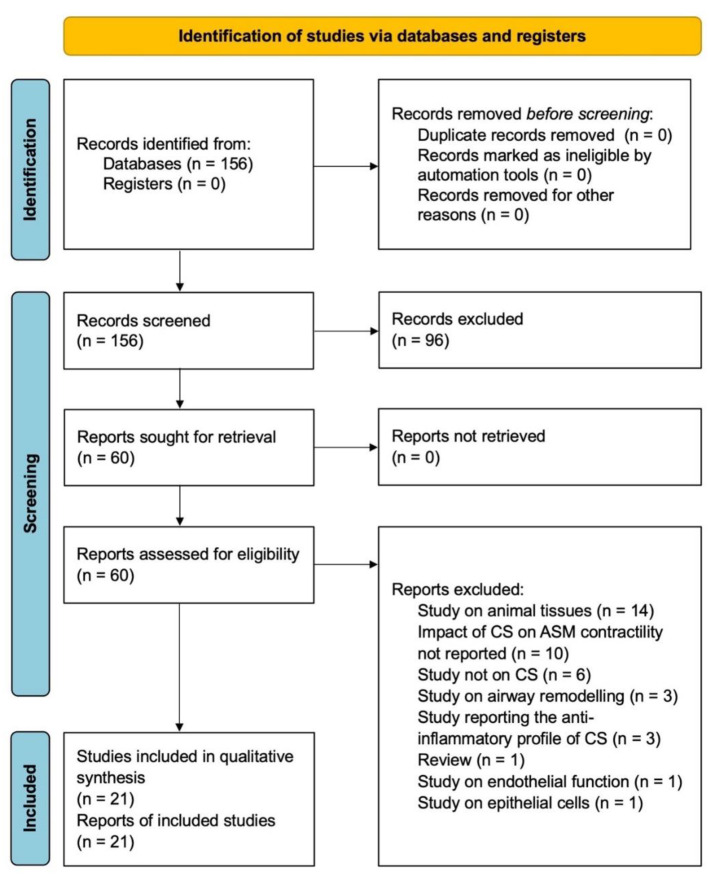
PRISMA 2020 flow diagram for the identification of the studies included in the systematic review. ASM: airway smooth muscle; CS: corticosteroid; and PRISMA: Preferred Reporting Items for Systematic Review and Meta-Analysis.

The protocol of this synthesis of the current literature was performed in agreement with the Preferred Reporting Items for Systematic Review and Meta-Analysis Protocols (PRISMA-P) [5], with the relative flow diagram shown in Figure 1. This study satisfied all the recommended items reported by the PRISMA 2020 checklist [6].

The PICO (Patient problem, Intervention, Comparison, and Outcome) framework was applied to develop the literature search strategy and question, as previously reported [7]. Namely, the “Patient problem” included increased ASM contractility or AHR; the “Intervention” regarded CS; the “Comparison” was performed with respect to the controls or the baseline; and the assessed “Outcome” was human ASM contractility or AHR that was not related to the anti-inflammatory effects of CS. 

A comprehensive literature search was performed for clinical studies, ex vivo studies, and in vitro studies written in English, characterizing the impact of CS on ASM contractility and AHR. The search was performed in MEDLINE in order to provide for relevant studies available with no time limit up to 25 July 2022. The research string was as follows: (“adrenal cortex hormones”[MeSH Terms] OR (“adrenal”[All Fields] AND “cortex”[All Fields] AND “hormones”[All Fields]) OR “adrenal cortex hormones”[All Fields] OR “corticosteroid”[All Fields] OR “corticosteroids”[All Fields] OR “corticosteroidal”[All Fields] OR “corticosteroide”[All Fields] OR “corticosteroides”[All Fields]) AND ((“airway”[All Fields] OR “airway s”[All Fields] OR “airways”[All Fields]) AND (“muscle, smooth”[MeSH Terms] OR (“muscle”[All Fields] AND “smooth”[All Fields]) OR “smooth muscle”[All Fields] OR (“smooth”[All Fields] AND “muscle”[All Fields]))) AND (“human s”[All Fields] OR “humans”[MeSH Terms] OR “humans”[All Fields] OR “human”[All Fields]) AND (“contractilities”[All Fields] OR “contractility”[All Fields] OR “muscle contraction”[MeSH Terms] OR (“muscle”[All Fields] AND “contraction”[All Fields]) OR “muscle contraction”[All Fields] OR “contractile”[All Fields] OR ((“airway”[All Fields] OR “airway s”[All Fields] OR “airways”[All Fields]) AND (“hyperresponsive”[All Fields] OR “hyperresponsiveness”[All Fields] OR “hyperresponsivity”[All Fields]))). 

Citations of previously published relevant and recently published reviews or editorials were examined to select further pertinent studies, if any [8]. Two reviewers independently checked the relevant studies identified from the literature search. The studies were selected in agreement with the previously mentioned criteria and any difference in opinion about eligibility was resolved by consensus. 

### 2.3. Data Extraction

Data from the included studies were extracted in agreement with Data Extraction for Complex Meta-anALysis (DECiMAL) recommendations [9] and checked for study references and year of publication, type of study, type of cells and tissue donors, characteristics of analyzed patients, contractile stimuli, number of tissue donors or patients, age and sex, treatments, route of administration, outcome measurements to evaluate the impact on ASM contractility and AHR, Jadad score, and Cochrane Risk of Bias (RoB).

### 2.4. Endpoints

The endpoint of this systematic review was the impact of CS on human ASM contractility and AHR that was not related to the anti-inflammatory effects of CS. The effects of CS were assessed both alone and in combination with long-acting β_2_-adrenoceptor agonists (LABA) and long-acting muscarinic antagonists (LAMA).

### 2.5. Strategy for Data Analysis

Data from original papers were extracted and reported via qualitative synthesis. 

### 2.6. Quality Score and RoB

The summary of the risk of bias for each included RCT was analyzed via the Jadad score [10] and Cochrane RoB 2 [11]. The Jadad score, with a scale of 1–5 (a score of 5 being the best quality), was used to assess the quality of the papers concerning the likelihood of bias related to randomization, double blinding, withdrawals and dropouts [10]. Studies were considered to be of low quality with a Jadad score <3, of medium quality with a Jadad score = 3, and of high quality with a Jadad score >3. The weighted assessment of the overall risk of bias was analyzed via the Cochrane RoB 2 tool [11] by using the robvis visualization software [12,13]. Two reviewers independently assessed the quality of individual studies, and any difference in opinion about the quality score was resolved by consensus. 

## 3. Results

### 3.1. Study Characteristics

Of the 155 potentially relevant records identified in the initial search, 21 studies were deemed eligible for a qualitative analysis. Data were obtained from eight studies conducted in vitro on human ASM cells (hASMC) [14,15,16,17,18,19,20] and lung myofibroblasts [21], seven ex vivo studies performed in human isolated bronchial tissue [4,22,23,24,25,26,27], and six clinical studies performed in asthmatic patients [28,29,30,31,32,33]. The following CS were investigated: beclomethasone dipropionate (BDP) [4,22,24,25,27], budesonide (BUD) [17,19,30,31,32], dexamethasone (DEX) [14,16,17,18,19,23], fluticasone propionate (FP) [14,19,20,21,28,29], mometasone furoate (MF) [26], and prednisolone (PSL) [15,33]. The main characteristics of the studies included in the systematic review are reported in Table 1.

### 3.2. Impact of CS Administered Alone In Vitro

#### 3.2.1. DEX

An in vitro study on hASMC investigated whether CS transcriptionally regulated the expression of CD38, a ~45-kDa glycosylated transmembrane protein having a central role in intracellular calcium homeostasis and AHR [18]. In hASMC transfected with a 3 kb human CD38 promoter containing a nuclear factor kappa-light-chain-enhancer of activated B cells (NF-κB), activator protein-1 (AP-1), and four GRE putative binding sites, 24 h treatment with DEX 1 μM completely reversed the two-fold activation of the promoter induced by tumor necrosis factor-alpha (TNF-α) 50 ng/mL [18]. 

Two-hour-pretreatment with DEX 1 μM significantly (*p* < 0.05) reduced the gene overexpression of CD38 in hASMC exposed to TNF-α 10 ng/mL (from 4.03 ± 0.33 to 1.21 ± 0.32 fold increase over basal) [19]. CD38 induction by costimulating TNF-α 10 ng/mL and interferon gamma (IFNγ) 500 IU/mL was insensitive to DEX [19]. 

A previous in vitro study [16] investigated the effect of DEX on inositol phosphate (IP) response produced by histamine (His) in primary cultured hASMC, considering the pivotal role of IP in inducing the release of calcium from intracellular stores with consequent ASM contraction [34]. When hASMC were preincubated with DEX at concentrations >10 nM for 22 h, the IP response to His 100 μM was significantly (*p* < 0.05) inhibited [16]. In hASMC stimulated with a range of His concentrations 1 μM–1 mM, pretreatment with DEX 1 μM significantly (*p* < 0.05) inhibited the formation of IP irrespective of His concentration. The effect of DEX was evident only after a preincubation of ≥6 h, and after 22 h (*p* < 0.001) the mean reduction observed was of 48.0 ± 5.0% [16]. 

Goldsmith et al. [14] investigated whether CS inhibit the expression of ASM contractile proteins in primary hASMC stimulated with transforming growth factor beta (TGFβ) 1 ng/mL. DEX 0.1 μM and 1 μM for 6 days significantly (*p* ≤ 0.01) reduced the overexpression of α-actin (from 7.12 ± 1.77-fold increase to 2.43 ± 0.49 and 2.47 ± 0.29-fold increase, respectively) and the short isoform of myosin light chain kinase (MLCK). Since DEX had no modulatory effect on the hASMC number, the authors concluded that a reduction in cell proliferation could not be the cause of the observed decrements in ASM protein abundance. The gene overexpression of α-actin, the rate of α-actin mRNA degradation, the synthesis of α-actin in presence of the transcriptional inhibitor actinomycin D, and the α-actin turnover were not modulated by DEX [14]. 

In hASMC stimulated by interleukin-1 beta (IL-1β) 10 U/mL to reproduce in vitro a cellular model of AHR, 3 h treatment with DEX 1 nM and 100 nM only numerically but not significantly (*p* > 0.05) reduced the gene overexpression of bradykinin B_2_ receptors [17]. One-hour-pretreatment with DEX 100 nM, but not 1 nM, significantly (*p* < 0.05) decreased the bradykinin 10 μM induced formation of IP after 6 h of incubation with IL-1β (from 12874.74 ± 739 dpm/assay to 8316.22 ± 1016 dpm/assay) [17].

#### 3.2.2. FP

In hASMC stimulated with TNF-α 10 ng/mL, pretreatment with FP 1–100 nM for 2 h dose-dependently suppressed CD38 gene overexpression, and the effect was significant (*p* < 0.05) at 50–100 nM concentrations [19]. Administering FP 100 nM to unstimulated hASMC did not affect the messenger ribonucleic acid (mRNA) level of CD38, while a significant (*p* < 0.05) reduction was observed when hASMC were exposed to TNF-α 10 ng/mL (from 5.00 ± 0.41 to 1.51 ± 0.33-fold increase over basal levels), IFNγ 500 IU/mL (from 1.95 ± 0.48 to 1.18 ± 0.40-fold increase over basal levels), and IFNβ (from 2.32 ± 0.49 to 1.02 ± 0.48-fold increase over basal levels) [19]. CD38 induction by co-stimulating TNF-α with IFNγ or IFNβ was insensitive to FP treatment [19]. 

In primary hASMC stimulated with TGFβ 1 ng/mL, FP 10 nM and 100 nM administered for 6 days significantly (*p* > 0.05) reduced the overexpression of α-actin (from 7.12 ± 1.77-fold increase to 2.34 ± 0.37 and 2.32 ± 0.66-fold increase, respectively, with FP at 10 nM and 100 nM) and reversed the shift in MLCK expression from the long to the short isoform [14]. Incubation for 48 h with FP did not affect (*p* > 0.05) the TGFβ induced-α-actin gene overexpression and the rate of mRNA degradation following the addition of the transcriptional inhibitor actinomycin D. Considering that α-actin was reduced at the protein level rather than at the mRNA level, the authors suggested a posttranscriptional control exerted by the CS [14]. In presence of actinomycin D, hASMC exposed to TGFβ for 24 h and incubated with FP showed a significant (*p* < 0.05) reduction in α-actin protein synthesis and improved α-actin protein turnover only when administered at 100 nM. FP decreased the TGFβ-induced incorporation of α-actin into filaments and significantly (*p* < 0.001) reduced cell length and contractile function in response to stimulation with acetylcholine (aCh) and potassium chloride (KCL) when administered at 1–100 nM concentrations [14]. 

Lewis et al. [20] evaluated spontaneous contraction in hASMC incubated alone or co-cultured with human lung mast cells (LMC). Incubation of hASMC or hASMC-LMC co-culture embedded in collagen gels with FP 10 μM for 16 h did not significantly (*p* > 0.05) affect the spontaneous contraction. 

In human bronchial myofibroblasts stimulated with TGFβ 5 ng/mL, 24 h incubation with FP 1 pM significantly (*p* ≤ 0.05) inhibited α-actin protein overexpression from 96.7 ± 1.5% to 29.3 ± 3.2% [21]. FP did not modulate the contractile activity of single myofibroblasts within 30 min of administration [21].

#### 3.2.3. BUD

In hASMC stimulated by IL-1β 10 U/mL to reproduce a cellular model of AHR in vitro, incubation with BUD 1 nM and 100 nM for 3 h numerically but not significantly (*p* > 0.05) reduced the gene overexpression of bradykinin B_2_ receptors [17]. When hASMC were pretreated with BUD 100 nM for 6 h, the synthesis of IP induced by bradykinin 10 μM was significantly (*p* < 0.05) reduced (from 12874.74 ± 739 dpm/assay to 6776.18 ± 585 dpm/assay) [17].

In hASMC stimulated by TNF-α 10 ng/mL, 2 h pretreatment with BUD 100 nM significantly (*p* < 0.05) lowered the gene overexpression of CD38 (from 4.02 ± 0.31 to 1.07 ± 0.43-fold increase over basal levels), but when cells were co-stimulated by TNF-α 10 ng/mL and IFNγ 500 IU/mL, no change was detected upon treatment with BUD [19].

#### 3.2.4. PSL

Goto et al. [15] investigated the effect of CS on the upregulation of the ras homolog family member A (RhoA), a guanosine-triphosphate-binding protein involved in calcium sensitization in antigen-induced AHR. In cultured hASMC, 24 h treatment with PSL 10 μM significantly (*p* < 0.001) inhibited the protein overexpression of RhoA induced by IL-13 100 ng/mL (from 1.29 ± 0.17 to 0.73 ± 0.03 [RhoA to β-actin ratio]) and TNF-α 10 ng/mL (from 0.84 ± 0.06 to 0.39 ± 0.03 [RhoA to β-actin ratio]). PSL significantly (*p* < 0.01) reduced RhoA promoter activity elicited by IL-13 and TNF-α [15].

### 3.3. Impact of CS Administered in Combination In Vitro

#### 3.3.1. DEX Plus LABA

In primary hASMC exposed to TGFβ 1 ng/mL, co-incubation with DEX 0.1–1 μM and salmeterol (SAL) 1 nM for 6 days significantly (*p* ≤ 0.01) reduced the overexpression of α-actin (from 7.12 ± 1.77 to a maximum of 3.05 ± 0.98) and the short isoform of MLCK [14]. Combining DEX with SAL did not result in a change in hASMC proliferation, therefore the authors argued against reduced cell number as the cause of the observed decrease in ASM proteins [14].

#### 3.3.2. FP Plus LABA

In primary hASMC exposed to TGFβ 1 ng/mL, co-incubation with FP 10 nM and SAL 1 nM for 6 days significantly (*p* ≤ 0.01) reduced the overexpression of α-actin from 7.12 ± 1.77 to 3.46 ± 1.02-fold increase, while FP 100 nM plus SAL induced a numerical decrease [14]. Adding SAL to FP 10–100 nM significantly (*p* ≤ 0.01) reversed the TGFβ-induced shift in MLCK expression from the long to the short isoform. Combining FP with SAL did not result in a change in hASMC proliferation [14].

In hASMC-embedded collagen gels, spontaneous contraction was significantly (*p* < 0.05) reduced when incubated for 16 h with FP 10 μM combined with formoterol (FOR) 1nM by ≃31.5% or with olodaterol (OLO) 1 nM by ≃36.3% [20]. When human hASMC were co-cultured with hLMC, FP 10 μM plus FOR or OLO significantly (*p* < 0.05) reduced spontaneous contraction by 23.2% and 38.9%, respectively [20].

In human bronchial myofibroblasts stimulated with TGFβ 5 ng/mL, co-incubation with FP 1 pM and SAL 10 nM for 24 h significantly (*p* ≤ 0.05) inhibited the protein overexpression of α-actin from 96.7 ± 1.5% to 2.3 ± 0.6% [21]. Within 30 min from FP and SAL administration, the contractile activity developed in single myofibroblasts disappeared in both young and aged cultures [21].

### 3.4. Impact of CS Administered Alone Ex Vivo

#### 3.4.1. DEX

One study [23] evaluated the impact of CS in precision cut lung slices (PCLS; small airways characterized by an inner diameter <2 mm) collected from non-asthma donors, incubated overnight with human immunoglobulin E (IgE), and stimulated by carbachol (CCh) 100 μM. Following cross-linking with high-affinity IgE receptor (FcεRI), overnight treatment with DEX 1 μM did not significantly (*p* > 0.05) modulate the FcεRI-dependent ASM contractility [23].

#### 3.4.2. BDP

In two ex vivo studies [22,25], overnight treatment with BDP 0.3–300 nM in medium bronchi and with BDP 1 nM–30 μM in PCLS did not significantly (*p* > 0.05) reduce the histaminergic contractile tone in non-sensitized tissues [22], in passively sensitized tissues incubated with serum from atopic asthma patients (total IgE 1000 U/mL) [22,25], and in airways collected from COPD donors and submaximally contracted by CCh [25].

Cazzola et al. [4] investigated the rapid non-genomic bronchorelaxant effect of BDP administered in medium bronchi and PCLS submaximally contracted with His. In non-sensitized medium bronchi, BDP modestly relaxed the histaminergic ASM tone in a concentration-dependent manner, reaching a maximal relaxant response (E_max_) of 33.41 ± 3.47% and a potency (expressed as the negative logarithm of the half-maximal effective concentration [pEC_50_]) of 4.79 ± 0.15. BDP was significantly (*p* < 0.001) more effective in passively sensitized medium bronchi, with an E_max_ of 43.61 ± 2.06% and a pEC_50_ of 5.09 ± 0.23. Pre-treatment with the Gsα subunit G protein antagonist NF449 and the cyclic-adenosine-monophosphate (cAMP)-dependent protein kinase A (PKA) inhibitor KT5720 significantly (*p* < 0.001) suppressed the non-genomic bronchorelaxant action of BDP in passively sensitized tissues, but not in non-sensitized ones, suggesting that the CS effect was dependent from the activation of Gsα–cAMP–PKA cascade [4]. In PCLS, the bronchorelaxant effect of BDP was significantly (*p* < 0.001) greater in passively sensitized tissues (E_max_ 63.89 ± 5.09% and pEC_50_ 7.23 ± 0.27) than in non-sensitized ones (E_max_ 31.94 ± 3.01% and pEC_50_ 7.27 ± 0.37) [4].

A recent ex vivo study [24] performed in medium bronchi contracted by transmural stimulation investigated whether pre-incubation with BDP for 1 h could abolish the AHR induced by cow’s milk (CM) aspiration, which has been implicated in the etiology of various inflammatory lung diseases. In tissues not challenged with CM, BDP 1–10 μM did not significantly (*p* > 0.05) modulate the ASM contractile tone. In airways challenged with CM 1:10 *v*/*v* for 60 min, BDP administered at 1 μM and 10 μM, but not at 0.1 μM, significantly (*p* < 0.05) lowered the ASM contractility by −52.49 ± 10.97% and −66.98 ± 7.90%, respectively [24].

### 3.5. Impact of CS Administered in Combination Ex Vivo

#### 3.5.1. BDP Plus LABA

Combining cumulative concentrations of BDP with FOR at a 100:6 concentration ratio induced a significant (*p* < 0.01) synergistic relaxant effect in medium bronchi pre-contracted by His, both in non-sensitized and passively sensitized tissues [22]. In non-sensitized medium bronchi, the maximal synergistic bronchorelaxant response was achieved with BDP/FOR 10/0.6 mg/mL and it was +28.73 ± 7.25% greater than the expected additive effect as predicted by the Bliss Independence equation, while in passively sensitized tissues the synergism remained stable over the range of concentrations 1/0.06–100/6 ng/mL, with a maximal synergistic bronchorelaxant response of +12.74 ± 4.62% compared to the additive effect [22]. A synergistic interaction was already observed at low concentrations of BDP/FOR inducing ≤25% of the E_max_, whereas for concentrations eliciting ≥50% E_max_ the extent of synergism was strong [22].

In non-sensitized and passively sensitized PCLS pre-contracted by His, increasing concentrations of BDP/FOR induced a significant (*p* < 0.001) synergistic bronchorelaxation. The maximal synergistic interaction was achieved by BDP/FOR 1/0.06 ng/mL in non-sensitized tissues (+20.41 ± 4.10% vs. expected additive effect) and by BDP/FOR 10/0.06 ng/mL in passively sensitized airways (+20.04 ± 2.18%). In non-sensitized PCLS, the synergistic interaction was greater at higher concentrations; the magnitude of interaction was strong at concentrations inducing 25–50% E_max_, and very strong at higher concentrations. In passively sensitized PCLS, BDP/FOR produced a greater synergistic interaction when administered at lower concentrations; the extent of synergism was very strong over the range of concentrations inducing 15–25% E_max_ and strong for concentrations inducing 75% E_max_ [22].

In medium bronchi collected from COPD donors, the concentration of BDP/FOR administered at a 100:6 concentration ratio that reduced by 50% the ASM contractility elicited by CCh (Ab EC_50_) was 2.31 ng/mL, while in PCLS, it was 4.59 ng/mL [27]. The potency of BDP/FOR was 1.56 ng/mL in medium bronchi and 0.97 ng/mL in PCLS. The combination effectively decreased the CCh-induced contractile tone in medium bronchi, reaching an E_max_ of 86.90%, whereas in PCLS the bronchorelaxant effect was only partial, with an E_max_ of 51.83% [27].

#### 3.5.2. MF Plus LABA

In passively sensitized medium bronchi pre-contracted by His, combining high, rather than medium concentrations of MF with the LABA indacaterol (IND) at a 100:90 molar ratio induced a significant (*p* < 0.05) bronchorelaxant effect and the maximal synergistic interaction was +17.61 ± 8.34% greater than the expected additive effect. The magnitude of synergistic interaction was strong to very strong [26]. In passively sensitized PCLS, combining medium concentrations of MF with IND at a 100:45 molar ratio elicited a significant (*p* < 0.05) bronchorelaxant effect on the histaminergic contractile tone, reaching an E_max_ +20.97 ± 7.47% greater than the expected additive effect, and the magnitude of synergistic interaction was very strong. When high concentrations of MF were combined with IND, the synergism significantly (*p* < 0.001) increased, reaching an E_max_ +27.36 ± 12.40% greater than the expected additive effect and the magnitude of interaction ranged from middle to very strong [26].

#### 3.5.3. BDP Plus LAMA

In non-sensitized medium bronchi and PCLS pre-contracted by His, BDP combined with glycopyrronium (GLY) at low concentrations of each monocomponent inducing 30% of the E_max_ (EC_30_) for 30 min did not induce synergism [4]. In passively sensitized medium bronchi, BDP/GLY elicited a synergistic bronchorelaxant effect on the histaminergic tone (+13.71 ± 1.60% vs. expected additive effect) and PCLS (+22.30 ± 5.39% vs. expected additive effect). When BDP/GLY was administered at low concentrations inducing EC_30_, the bronchorelaxant response was 64.71 ± 1.60% in passively sensitized medium bronchi and 73.30 ± 5.39% in PCLS [4].

#### 3.5.4. Triple Combinations Including BDP

In an ex vivo model of bronchial asthma, combining BDP with the LABA FOR, and the LAMA GLY at a 100:6:12.5 concentration ratio produced a significant (*p* < 0.05) synergistic bronchorelaxant effect in passively sensitized medium bronchi and PCLS submaximally contracted by His [25]. The maximal synergistic interaction was achieved with BDP/FOR/GLY 1/0.06/0.125 ng/mL (+43.57 ± 0.96% vs. expected additive effect). The extent of synergism was very strong and overall stable across the range of concentrations inducing 25–90% E_max_. In passively sensitized PCLS, the maximal synergistic interaction was achieved with BDP/FOR/GLY 10/0.6/1.25 ng/mL (+24.95 ± 7.85% vs. expected additive effect). When administered at concentrations inducing 25–75% E_max_, BDP/FOR/GLY produced a very strong synergism, while at concentrations inducing 90% E_max_, synergism was strong [25].

In an ex vivo model of stable COPD [25], BDP/FOR/GLY administered at a 100:6:12.5 concentration ratio elicited a synergistic interaction in medium bronchi and PCLS submaximally contracted by CCh. In medium bronchi, the maximal synergistic bronchorelaxant response was detected with BDP/FOR/GLY 1/0.06/0.125 μg/mL (+51.64 ± 4.41% vs. additive effect), while in PCLS with BDP/FOR/GLY 3/0.18/0.375 ng/mL (+28.85 ± 5.01% vs. additive effect) [25]. In medium bronchi, BDP/FOR/GLY administered at concentrations eliciting 25–90% E_max_ elicited a stable very strong synergistic interaction, whereas in PCLS the synergism was low at concentrations inducing 25% E_max_, strong at concentrations inducing 50% E_max_, and very strong at concentrations eliciting ≥75% E_max_ [25].

A more recent ex vivo study [27] confirmed that in medium bronchi collected from COPD donors and submaximally contracted by CCh, the triple BDP/FOR/GLY combination administered at a 100:6:12.5 concentration ratio produced a significant (*p* < 0.05) synergistic bronchorelaxant effect. Synergism was observed with BDP/FOR administered at concentrations of 0.318–31.8 ng/mL plus GLY at concentrations of 0.0375–3.75 ng/mL. The maximal synergistic interaction was reached when BDP/FOR 1.06 ng/mL was combined with GLY 0.125 ng/mL, reaching an improved bronchorelaxation of +32.00–35.00%, compared to the expected additive effect [27].

In PCLS collected from COPD donors and submaximally contracted with CCh, BDP/FOR/GLY administered at a 100:6:12.5 concentration ratio induced a supra-additive effect, as resulted from the analysis of interaction via Bliss model; according to the Loewe and HSA models, a significant (*p* < 0.05) synergistic bronchorelaxant response resulted when BDP/FOR administered at concentrations of 1.06–31.8 ng/mL was combined with GLY at concentrations of 0.125–3.75 ng/mL. Maximal synergism was detected for BDP/FOR 10.6 ng/mL combined with GLY 1.25 ng/mL, leading to an improved bronchorelaxant effect of +36.00–37.00%, compared to the expected additive effect [27].

#### 3.5.5. Triple Combinations Including MF

In passively sensitized medium bronchi and PCLS pre-contracted by His, combining medium concentrations of MF with the LABA IND and the LAMA GLY at a 100:37:45 molar ratio produced a significant (*p* < 0.05) synergistic bronchorelaxant effect and the E_max_ achieved was +22.94 ± 13.81% greater than the additive effect [26]. Synergism was further significantly (*p* < 0.001) increased when high MF concentrations were combined with IND and GLY at a 100:37:90 molar ratio, and the E_max_ achieved was +28.73 ± 2.59% greater than the additive effect. The magnitude of synergistic interaction was always very strong, irrespective of MF concentration. In passively sensitized PCLS, treatment with medium concentrations of MF, IND, and GLY at a 100:37:45 molar ratio produced a significant (*p* < 0.001) synergistic bronchorelaxant response, reaching an E_max_ +45.00 ± 4.41% greater than the additive effect. The synergistic interaction was further significantly (*p* < 0.001) enhanced when high concentrations of MF were combined with IND and GLY at the 100:37:90 molar ratio, and the E_max_ was +53.72 ± 9.10% compared with the expected additive effect. The magnitude of synergistic interaction was always very strong, irrespective of MF concentration [26].

### 3.6. Impact of CS Administered Alone in Clinical Studies

#### 3.6.1. FP

Currie et al. [29] performed an RCT in mild asthmatic patients to characterize the impact of FP 250 μg twice daily (BID) on AHR, defined by the methacholine (MCh) challenge test. Treatment with FP for 3 weeks significantly (*p* < 0.05) increased the MCh provocative dose causing a 20% fall in FEV_1_ (PD_20_), by producing a doubling dose improvement of 1.6 (95% CI 0.8–2.3) vs. the baseline [29].

In another RCT [28], two-weeks treatment with FP 500 μg BID significantly (*p* < 0.01) increased the provocative concentration causing a 20% decrease in FEV_1_ (PC_20_) to MCh vs. the baseline (2.50-doubling dilutions [dd] shift, 95% CI 1.43–3.26) in non-smoking asthmatic patients. In contrast, FP was not significantly (*p* > 0.05) effective on AHR in patients who smoke. The response to FP was significantly (*p* < 0.01) different between current smokers and non-smokers (2.54-dd shift, 95% CI 1.51–3.56) [28].

#### 3.6.2. BUD

O’Connor et al. [31] investigated the effect of AMP and sodium metabisulfite (MBS) in mild asthmatic patients. Subjects underwent a bronchoprovocation challenge with inhaled AMP, MBS, and MCh before and after 2 weeks of treatment with BUD 0.8 mg BID [31]. Compared to the placebo, BUD significantly (*p* < 0.01) reduced the AHR to MBS and MCh to a similar extent, shifting the dose–response curve of each agonist to the right by 1.06-dd (0.34–1.78) and 1.17-dd (95% CI 0.34–2.00), respectively. BUD induced a further significant (*p* < 0.01) reduction in the AHR to AMP vs. placebo and the other challenges, shifting rightward the dose–response curve by 2.92-dd (2.12–3.72) [31].

Kelly et al. [30] conducted an RCT to evaluate the effect of 11 days of treatment with BUD on AHR in patients with mild atopic asthma, before and after an allergen inhalation challenge at day 9. Compared to the baseline, BUD 400 μg BID significantly (*p* < 0.05) increased the PC_20_ to MCh at day 8 of treatment pre-allergen challenge and prevented the allergen-induced AHR at day 11 of treatment.

According to a clinical study [32] performed on patients with mild asthma, 4 weeks of treatment with BUD 200 μg BID significantly (*p* < 0.05) increased the PC_20_ to MCh from 3.7 ± 2.7 mg/mL to >16 mg/mL vs. the baseline.

#### 3.6.3. PSL

Yick et al. [33] evaluated the effect of 2 weeks of oral treatment with PSL 0.5 mg/kg/day on AHR and investigated changes in the ASM transcriptomic profile in endobronchial biopsies of patients with atopic asthma. PSL numerically but not significantly (*p* > 0.05) increased MCh PC_20_ vs. the baseline and vs. the placebo. Across the 15 genes modulated by treatment with PSL, the FAM129A and SYNPO2 genes resulted to be significantly (*p* < 0.01) correlated with AHR (r = −0.740 and r = −0.746, respectively) [33].

### 3.7. RoB and Quality of Evidence

**Figure 2:**
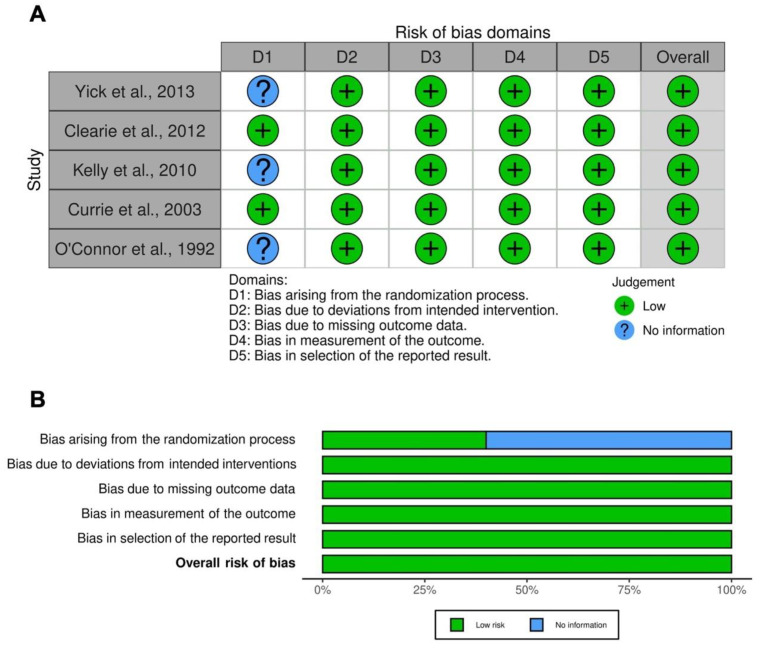
Traffic light plot for the assessment of the risk of bias of each included randomized trial (**A**) and weighted plot for the assessment of the overall risk of bias via the Cochrane RoB 2 tool (**B**) (*n* = 5 studies). Traffic light plot reports five risk-of-bias domains: D1, bias arising from the randomization process; D2, bias due to deviations from the intended intervention; D3, bias due to missing outcome data; D4, bias in measurement of the outcome; and D5, bias in the selection of the reported result; a green circle represents a low risk of bias and a blue circle indicates insufficient information on the risk of bias. RoB: risk of bias [28,29,30,31,33].

Of the six clinical studies [28,29,30,31,32,33] included in the systematic review, five RCTs [28,29,30,31,33] were assessable via the Cochrane RoB 2, whilst the study by Williams et al. [32] was neither randomized nor controlled, therefore it was not feasible for RoB judgement. All the studies (five, 100.0%) had a low risk of bias for deviations from intended interventions, missing outcome data, measurement of the outcome, and selection of the reported result. Three RCTs (60.0%) did not report information for the RoB in the randomization process, and the other two studies (40.0%) presented a low risk of bias. The overall RoB was low for all the included RCTs (five, 100.0%). Detailed information concerning the RoB assessment is reported in Figure 2. All the included RCTs were ranked as being of medium quality, in agreement with the Jadad score (Table 1).

## 4. Discussion

**Figure 3:**
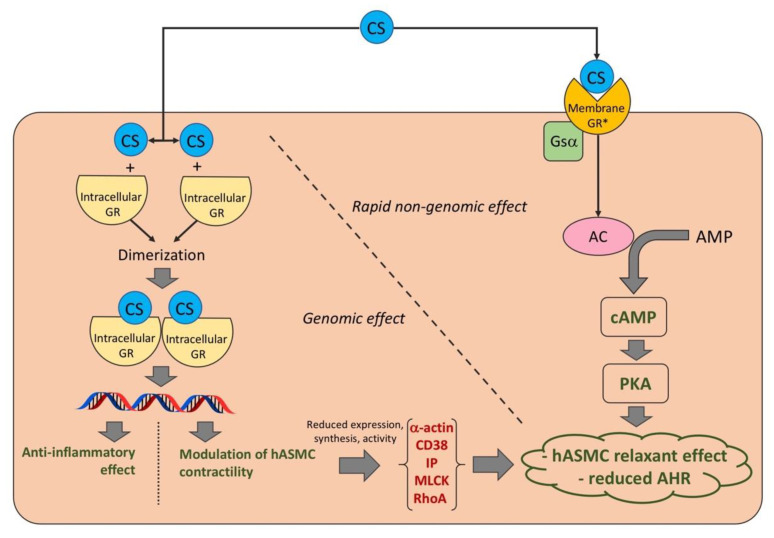
Genomic and rapid non-genomic effect of CS on human ASM contractility and AHR. * Putative non-classic membrane receptors characterized by pharmacological properties that are different from those of classic intracellular GR. AC: adenylyl cyclase; AHR: airway hyperresponsiveness; AMP: adenosine monophosphate; cAMP: cyclic adenosine monophosphate; CS: corticosteroids; GR: glucocorticoid receptors; hASMC: human airway smooth muscle cells; IP: inositol phosphate; MLCK: myosin light chain kinase; PKA: protein kinase A; and RhoA: ras homolog family member A.

The findings resulting from this systematic review indicate that, generally, CS have significant genomic/non-genomic beneficial effects on human ASM contractility and AHR, regardless of their anti-inflammatory effects (Figure 3).

More specifically, CS are effective in vitro in reducing either the expression, synthesis or activity of α-actin, CD38, IP, MLCK, and RhoA in response to several stimuli (i.e., ACh, bradykinin, IL-13, KCL, TGFβ, and TNF-α) that increase the contractile tone of hASMC [35,36,37]; overall these effects were mediated by the genomic action of CS. Moreover, CS have been demonstrated to elicit strong bronchorelaxant effects in both small and medium human isolated airways via the rapid activation of the Gsα–cAMP–PKA pathway after a passive sensitization procedure, which is a validated model mimicking ex vivo the hyperreactivity of asthmatic bronchial tissue [38]. Interestingly, data originating from ex vivo studies were corroborated by clinical studies [28,29,30,31,32] carried out in mild asthmatic patients, reporting that CS are effective in reducing or preventing AHR elicited by different pro-contractile stimuli (i.e., AMP, MBS, and MCh). A summary of the beneficial effects of specific CS, alone or in combination with bronchodilators, against human ASM contractility and AHR in vitro, ex vivo, and in clinical trials is reported in Table 2 and Table 3.

The evidence that, when administered in combination with a LABA, CS induced significant synergistic bronchorelaxant effects in passively sensitized small and medium human bronchi fully supports the current global initiative for asthma (GINA, 2022) approach in which an ICS are recommended for Steps 1–5 [39]. Effectively, in this range of treatments, ICS-FOR was suggested as the preferred controller and reliever therapy by modulating the dose of ICS in the fixed-dose combination (FDC) according to the disease severity [39]. To date ICS, always combined with FOR, represents the cornerstone of asthma treatment not only as a maintenance therapy, but also as-needed for the relief of symptoms and, if needed, before exercise [39]. Of note, this pharmacological approach reduces the risk of exacerbation compared with using a short-acting β_2_-adrenoceptor agonist (SABA) reliever [39].

ICS-FOR as a MAintenance and Reliever Therapy (MART), currently recommended at Step 3–5 [39], resulted in acute and dose-related anti-inflammatory effect in symptomatic asthmatic patients [40]. Interestingly, in the same patients and in an acute setting, high-dose MART also exerted significant improvements in FEV_1_ compared to the SABA terbutaline [40].

Triple ICS/LABA/LAMA FDC is currently considered an alternative treatment at Step 4 as well as a preferred therapy at Step 5 [39]. As a matter of fact, a strong to very strong synergistic interaction has been proved ex vivo among ICS, LABA, and LAMA [25,26]. Furthermore, triple ICS/LABA/LAMA FDC exerted ceiling bronchorelaxation at the level of small airways in humans, by improving hyperinflation in more severe patients and leading to substantial clinical benefits [27]. Therefore, there is the pharmacological rationale for combining an ICS with a LABA plus a LAMA, both characterized by a rapid bronchorelaxant onset and long duration such as FOR plus GLY [41,42,43], and administered as a Triple MAintenance and Reliever Therapy (TriMART). The possibility of modulating the dose of the ICS in the triple BDP/FOR/GLY FDC (lower dose 100/6/12.5 µg, higher dose 200/6/12.5 µg) [44] makes the novel TriMART approach a potential therapeutic strategy for asthmatic patients at Step 3–5, especially for those subjects who may benefit from a sustained bronchodilation and suffering from increased parasympathetic tone [45].

Another important point arising from this systematic review is that ICS can be ineffective in preventing AHR in smoking patients with asthma, a condition related to the presence of relative steroid resistance due to the impairment of histone deacetylase 2 (HDAC2) [28,46]. In these smoking asthmatics, perhaps adding drugs that are able to restore HDAC2 activity such as doxofylline may help support the therapeutic effect of ICS [47,48,49,50,51].

This systematic review has certainly some limitations, generally intrinsic to primary publications. First, although the large body of evidence resulting from in vitro and ex vivo research supported the acute direct effects of ICS against human ASM contractility and AHR, ad-hoc translational studies are still missing for most of the ICS. However, we can speculate that the beneficial effects of ICS on airway contractility is an effect of class not specific for each single molecule. Second, the trial by Williams et al. [32] was not an RCT, therefore it was not possible to assess the quality of this clinical study, whose results should be interpreted with caution. Third, the remaining RCTs [28,29,30,31,33] included in this systematic review were proof-of-concept studies that enrolled a small number of patients. Regrettably to date, it seems that excluded the evident interest raised from ex vivo research; the direct impact of ICS on ASM contractility and AHR is no longer a hot topic in clinical research in asthma and COPD.

In conclusion, while the genomic effects of CS have been well-characterized with respect to the expression, synthesis, and activity of pro-contractile factors, current evidence suggests that CS may also elicit rapid non-genomic effects on human airways via the Gsα–cAMP–PKA pathway, a cascade activated by one or more specific CS membrane-associated receptors [52,53]. However, since the existence of such distinct non-classic CS membrane receptors has been not yet proven [3], further studies are needed to demonstrate the expression of these putative receptors on hASMC.

## Figures and Tables

**Table 1:** Characteristics of the studies included in the systematic review.

Study and Year	Type of Study	Type of Human Cells, Tissue Donors, or Characteristics of Analyzed Patients	Contractile Stimulus (Dose)	Number of Patients or Tissue Donors	Age	Male (%)	CS with Dose (Exposure Time or Treatment Duration)	Route of Administration	Jadad Score	Investigated Outcomes
Ritondo et al., 2021 [24]	Ex vivo study	Medium bronchi from patients undergoing lobectomy surgery for lung cancer	Cow’s milk (1:10 *v*/*v*)	16	50.0	50.0	BDP 0.1–10 μM (1 hr)	Incubation	/	Bronchorelaxant effect
Rogliani et al., 2021 [26]	Ex vivo study	Medium bronchi and PCLS from patients undergoing surgery for lung cancer with normal lung function and without history of chronic airway disease (tissues were passively sensitized with serum from atopic asthma patients)	His in passively sensitized tissues (EC_70_); CCh in COPD tissues (EC_70_)	13	50.0	50.0	MF/IND at 100:45 or 100:90 concentration-ratio (cumulative concentrations); MF/IND/GLY at 100:37:45 or 100:37:90 concentration-ratio	Incubation	/	Bronchorelaxant effect and pharmacological interaction
Rogliani et al., 2021 [27]	Ex vivo study	Medium bronchi and PCLS from COPD donors	CCh in COPD tissues (EC_70_)	16	69.3	93.8	BDP/FOR at 100:6 concentration-ratio (cumulative concentration); BDP/FOR/GLY at 100:6:12.5 concentration-ratio (cumulative concentrations)	Incubation	/	Bronchorelaxant effect and pharmacological interaction
Rogliani et al., 2020 [25]	Ex vivo study	Medium bronchi and PCLS from patients undergoing surgery for lung cancer with normal lung function and without history of chronic airway disease (asthma model, tissues were passively sensitized with serum from atopic asthma patients); medium bronchi and PCLS from COPD donors with a lung function in agreement with spirometric diagnosis of COPD FEV_1_/FVC < 0.7 (COPD model)	His in passively sensitized tissues (EC_70_); CCh in COPD tissues (EC_70_)	32	50.4	53.1	BDP 0.3–300 nM (overnight); BDP/FOR/GLY at 100:6:12.5 concentration-ratio (cumulative concentrations)	Incubation	/	Bronchorelaxant effect and pharmacological interaction
Calzetta et al., 2018 [22]	Ex vivo study	Medium bronchi and PCLS from patients undergoing lobectomy surgery for lung cancer, without a history of chronic airway disease; tissues were non-sensitized (incubated with serum from non-atopic donors) or passively sensitized (with serum from atopic asthma patients)	His (EC_70_)	16	50.0	50.0	BDP 1–100 nM (overnight); BDP/FOR at 100:5 concentration-ratio (cumulative concentrations)	Incubation	/	Bronchorelaxant effect and pharmacological interaction
Cazzola et al., 2016 [4]	Ex vivo study	Medium bronchi and PCLS from patients undergoing lobectomy surgery for lung cancer, without a history of chronic airway disease; tissues were non-sensitized (incubated with serum from non-atopic donors) or passively sensitized (with serum from atopic asthma patients)	His (EC_70_)	14	63.3	57.1	BDP (0.1 nM–10 μM); BDP/GLY at concentrations inducing EC_30_	Incubation	/	Bronchorelaxant effect and pharmacological interaction
Koziol-White et al., 2016 [23]	Ex vivo study	PCLS from non-asthmatic donors	CCh (100 μM)	11	43.2	72.7	DEX 1 μM (overnight)	Incubation	/	FcεRI-cross-linking-induced ASM contraction
Lewis et al., 2015 [20]	In vitro study	hASMC from asthmatic and healthy donors, cultured alone or co-cultured with hLMC	Not present	≃6	NA	NA	FP 10 μM; FP 10 μM + FOR 1 nM or OLO 1 nM	Incubation	/	Spontaneous contraction in collagen gel assay
Yick et al., 2013 [33]	Double-blind, randomized, PCB-controlled, parallel study	Atopic asthma patients (non-smoking or stopped for >12 months with smoking history of <5 pack-years; no exacerbations within 6 weeks before participation; steroid-naive or stopped using CS by any dosing route for ≥8 wks before participation; MCh PC_20_ ≤ 8 mg/mL; post-bronchodilator FEV_1_ >70% predicted)	MCh (NA)	12	24.5	NA	PSL 0.5 mg/kg/day	Oral	3	Methacholine PC_20_
Clearie et al., 2012 [28]	Single-center, double-blind, crossover, randomized, PCB-controlled study	Mild to moderate persistent asthma patients (FEV_1_ ≥ 60% [<30% PEF variability], prescribed ≤1000 μg BDP or equivalent)	MCh (NA)	31	38.4	48.4	FP 500 μg BID (2 wks); FP/SAL 250/50 μg BID (2 wks)	Oral inhalation (pMDI)	3	MCh PC_20_
Goto et al., 2010 [15]	In vitro study	Cultured hASMC	IL-13 (100 ng/mL), TNF-α (10 ng/mL)	NA	NA	NA	Prednisolone 10 μM (24 hrs)	Incubation	/	RhoA protein expression and RhoA promoter activity
Kelly et al., 2010 [30]	Prospective, double-blind, crossover, randomized, PCB-controlled study	Mild atopic asthma patients (MCh PC_20_ < 16 mg/mL and allergen-induced early and late bronchoconstrictor responses of ≥15% reduction in FEV_1_ during screening challenge)	MCh (NA)	14	26.0	42.9	BUD 400 μg BID (11 days); BUD/FOR 400/12 μg BID (11 days); PCB BID	Oral inhalation (DPI)	3	MCh PC_20_
Williams et al., 2008 [32]	Clinical study	Mild asthma patients (history of intermittent wheeze, treatment with albutamol inhaler on an intermittent basis, CS-naive, with positive skin-prick test to common aeroallergens)	MCh (NA)	5	29.0	12.5	BUD 200 μg BID (4 wks)	Oral inhalation (DPI)	/	MCh PC_20_
Tirumurugaan et al., 2008 [18]	In vitro	Cultured hASMC from donors	TNF-α (50 ng/mL)	3	NA	NA	DEX 1 μM (24 hrs)	Incubation	/	CD38 gene expression
Goldsmith et al., 2007 [14]	In vitro	Primary hASMC	TGFβ (1 ng/mL)	NA	NA	NA	DEX 0.1,1 μM (6 days); DEX 0.1,1 μM + SAL 1 nM (6 days); FP 10, 100 nM (48 hrs, 6 days); FP 10 nM + SAL 1 nM (6 days)	Incubation	/	Gene and protein expression of α-actin, protein expression of MLCK, rate of α-actin mRNA degradation, synthesis of α-actin in presence of actinomycin D, α-actin turnover, and contractile response to aCh and KCl-induced stimulation
Tliba et al., 2006 [19]	In vitro	hASMC from lung transplant donors	TNF-α (10 mg/mL), IFNγ (500 IU/mL), IFNβ (500 IU/mL)	NA	NA	NA	DEX 1 μM (2 hrs); FP 1, 10, 50, 100 nM (2 hrs); BUD 100 nM (2 hrs)	Incubation	/	CD38 gene overexpression
Baouz et al., 2005 [21]	In vitro	Myofibroblasts	TGFβ (5 ng/mL)	NA	NA	NA	FP 1 pM (24 hrs); FP 1 pM + SAL 10 nM (24 hrs)	Incubation	/	α-actin protein expression and contractile activity of single myofibroblasts evaluated within 30 min from treatment administration
Currie et al., 2003 [29]	Single-center, double-blind, crossover, randomized study	Mild asthma patients (FEV_1_ > 80% predicted and MCh PD20 < 500 μg)	MCh (NA)	14	21.4	36.0	FP 250 μg BID (3 wks); FP/SAL 125/25 μg BID (3 wks)	Oral inhalation (pMDI)	3	MCh PD_20_
Schmidilin et al., 1998 [17]	In vitro	Primary hASMC	IL-1β (10 U/mL)	NA	NA	NA	DEX 1, 100 nM (1 hr); BUD 1, 100 nM (3 hrs, 6 hrs)	Incubation	/	Gene overexpression of bradykinin B_2_ receptor, and IP synthesis
Hardy et al., 1996 [16]	In vitro	Primary hASMC	His (100 μM or range of concentrations 1 μM–1 mM)	NA	NA	NA	DEX 1 nM–1 μM (1–22 hrs)	Incubation	/	IP response and synthesis
O’Connor et al., 1992 [31]	Randomized, double-blind, PCB-controlled, crossover study	Mild atopic asthma patients	AMP, MBS, MCh (NA)	NA	NA	NA	BUD 0.8 mg BID (2 wks)	Oral inhalation	3	PC_20_ to AMP, MBS, MCh

aCh: acetylcholine; AMP: adenosine monophosphate; BDP: beclomethasone dipropionate; BID: twice daily; BUD: budesonide; CCh: carbachol; COPD: chronic obstructive pulmonary disease; CS: corticosteroids; DEX: dexamethasone; DPI: dry powder inhaler; EC_30_: concentration inducing 30% of the maximal effect; EC_70_: concentration inducing 70% of the maximal effect; FEV_1_: forced expiratory volume in the first second; FOR: formoterol fumarate; FP: fluticasone propionate; FVC: forced vital capacity; GLY: glycopyrronium; hASMC: human airway smooth muscle cells; hLMC: human lung mast cells; His: histamine; hrs: hours; IL-n: interleukin-n; IP: inositol phosphate; KCl: potassium chloride; MBS: sodium metabisulfite; MCh: methacholine; MF: mometasone furoate; MLCK: myosin light chain kinase; mRNA: messenger ribonucleic acid; NA: not available; OLO: olodaterol; PC_20_: provocative concentration causing 20% decrease in FEV_1_; PCB: placebo; PCLS: precision cut lung slices; PD_20_: provocative dose causing 20% fall in FEV_1_; pMDI: pressurized metered dose inhaler; PSL: prednisolone; RhoA: ras homolog family member A; SAL: salmeterol; TGFβ: transforming growth factor beta; TNF-α: tumor necrosis factor-alpha; and wks: weeks.

**Table 2:** Statistically significant (*p* < 0.05) effects of specific CS administered as monocomponents against human ASM contractility and AHR in vitro, ex vivo, and in clinical trials as resulting from this systematic review.

	Corticosteroids Administered as Monocomponents				
Experimental Setting	BDP	BUD	DEX	FP	PSL
hASMC (in vitro)	NA	↓ CD38 overexpression to TNF-α ↓ IP synthesis to bradykinin	↓ CD38 overexpression to TNF-α ↓ IP synthesis to His ↓ α-actin overexpression to TGFβ ↓ short isoform of MLCK overexpression to TGFβ	↓ CD38 overexpression to TNF-α ↓ α-actin overexpression to TGFβ ↑ α-actin protein turnover to TGFβ ↓ α-actin incorporation into filaments, reduced cell length and contractility to ACh and KCL Reversed the shift in MLCK expression from the long to the short isoform	↓ RhoA overexpression to IL-13 ↓ RhoA overexpression to TNF-α
Human myofibroblasts (in vitro)	NA	NA	NA	↓ α-actin overexpression to TGFβ	NA
Human medium bronchi (ex vivo)	Weak relaxant effect to His in non-sensitized tissue Strong relaxant effect to His in passively sensitized tissue ↑ Gsα–cAMP–PKA cascade in passively sensitized tissue ↓ contractility to EFS in cow’s milk challenged tissue	NA	NA	NA	NA
Human PCLS (ex vivo)	Weak relaxant effect to His in non-sensitized tissue Strong relaxant effect to His in passively sensitized tissue	NA	NA	NA	NA
Mild asthmatic patients (clinical trials)	NA	↓ AHR to MBS ↓ AHR to MCh ↓ AHR to AMP Improvement in PD_20_ to MCh, also post allergen challenge Prevention of allergen-induced AHR	NA	Improvement in PD_20_ to MCh (greater effect in non-smokers than in current smokers)	Correlation of FAM129A and SYNPO2 genes with AHR

↑: increase; ↓: reduction; ACh: acetylcholine; AHR: airway hyperresponsiveness; AMP: adenosine monophosphate; BDP: beclomethasone dipropionate; BUD: budesonide; DEX: dexamethasone; FP: fluticasone propionate; hASMC: human airway smooth muscle cells; His: histamine; IL-n: interleukin-n; IP: inositol phosphate; KCl: potassium chloride; MBS: sodium metabisulfite; MCh: methacholine; MLCK: myosin light chain kinase; NA: not available; PD_20_: provocative dose causing a 20% fall in FEV_1_; PSL: prednisolone; RhoA: ras homolog family member A; TGFβ: transforming growth factor beta; and TNF-α: tumor necrosis factor-alpha.

**Table 3:** Statistically significant (*p* < 0.05) effects of specific CS administered in dual and triple combinations with bronchodilators against human ASM contractility and AHR in vitro and ex vivo studies as resulting from this systematic review.

	Corticosteroids Administered in Combination with Bronchodilators							
Experimental Setting	BDP + LABA	BDP + LAMA	BDP + LABA + LAMA	DEX + LABA	FP + LABA	FP + LAMA	MF + LABA	MF + LABA + LAMA
hASMC (in vitro)	NA	NA	NA	↓ α-actin overexpression to TGFβ ↓ short isoform of MLCK to TGFβ	↓ α-actin overexpression to TGFβ ↓ short isoform of MLCK to TGFβ ↓ spontaneous contractility	↓ spontaneous contractility	NA	NA
Human myofibroblasts (in vitro)	NA	NA	NA	NA	↓ α-actin overexpression to TGFβ	NA	NA	NA
Human medium bronchi (ex vivo)	Strong synergistic relaxant effect to His in passively sensitized tissue ↓ CCh-induced contractile tone in tissue from COPD donors	Synergistic relaxant effect to His in passively sensitized tissue	Very strong synergistic relaxant effect to His in passively sensitized tissue Very strong synergistic relaxant effect to CCh in tissue from COPD donors	NA	NA	NA	Strong to very strong synergistic relaxant effect to His in passively sensitized tissue	Very strong synergistic relaxant effect to His in passively sensitized tissue
Human PCLS (ex vivo)	Very strong synergistic relaxant effect to His in passively sensitized tissue ↓ CCh-induced contractile tone in tissue from COPD donors	Synergistic relaxant effect to His in passively sensitized tissue	Very strong synergistic relaxant effect to His in passively sensitized tissue Strong to very strong synergistic relaxant effect to CCh in tissue from COPD donors	NA	NA	NA	Mild to very strong synergistic relaxant effect to His in passively sensitized tissue	Very strong synergistic relaxant effect to His in passively sensitized tissue

↓: reduction; AHR: airway hyperresponsiveness; BDP: beclomethasone dipropionate; BUD: budesonide; COPD: chronic obstructive pulmonary disease; CCh: carbachol; DEX: dexamethasone; FP: fluticasone propionate; hASMC: human airway smooth muscle cells; His: histamine; LABA: long-acting β_2_-adrenoceptor agonists; LAMA: long-acting muscarinic antagonists; MF: mometasone furoate; MLCK: myosin light chain kinase; NA: not available; and TGFβ: transforming growth factor beta.

## Data Availability

The data presented in this study are available in the article.
